# Laboratory and Clinical Efficacy of 6% Hydrogen Peroxide At-Home Whitening Systems with Custom Trays

**DOI:** 10.3390/bioengineering13070815

**Published:** 2026-07-16

**Authors:** Mayumi Maesako, Nagisa Matsui, Miyuki Oshika, Masao Irie, Akimasa Tsujimoto

**Affiliations:** 1 Department of Operative Dentistry, Aichi Gakuin University School of Dentistry, Nagoya 464-8651, Japan; maesako@dpc.agu.ac.jp (M.M.); ag243d14@az.agu.ac.jp (N.M.);; 2 Department of Occlusal & Oral Functional Rehabilitation, Okayama University Graduate School of Medicine, Dentistry and Pharmaceutical Sciences, Okayama 700-8558, Japan; 3 Department of Operative Dentistry, The University of Iowa College of Dentistry and Dental Clinics, Iowa City, IA 52242, USA; 4 Department of General Dentistry, Creighton University School of Dentistry, Omaha, NE 68102, USA

**Keywords:** whitening, hydrogen peroxide, enamel

## Abstract

Demand for fast and convenient dental whitening has increased in recent years. This paper compares the efficacy of three home whitening systems (TiON Take Home with (W), Opalescence Go (G), and Angelus Home 16% (A)) and presents a clinical case. W and G contain 6% hydrogen peroxide, while A contains 16% carbamide peroxide, approximately equal active peroxide contents. The whitening agents were applied to brown chicken eggshells for 90 (W90, G90, A90) or 60 (W60) minute periods ten (W90, W60, G90) or fourteen (A90) times. The color was measured before treatment, and after 3, 5, 7, 10, 12, and 14 applications. ∆E*_ab_ was calculated at each point. ∆E*_ab_ at completion were 4.97 (A90), 7.25 (G90), 9.84 (W60), and 14.96 (W90). A clinical study of the efficacy of W60 found a ∆E*_ab_ of 4.18. The whitening efficacy of a system is not determined entirely by the content of active peroxide.

## 1. Introduction

In contemporary dental practice, demand from patients for esthetic treatment is increasing [1]. People now seek a higher level of whitening than in the past, which has been linked to the spread of SNS and to the increased use of video conferencing during and after the pandemic [2]. This trend is broad, being seen in many countries and also extending beyond dentistry to minor forms of cosmetic surgery. This development raises ethical concerns about the risk of over-treatment, and it is important that dentists consider the broader impact of procedures on the patient’s health and well-being [3].

In this context, dental whitening has become the treatment of choice for addressing tooth discoloration, due to its non-invasive nature and relatively low cost compared to other invasive esthetic restorative treatments [4]. Various types of tooth discoloration can affect the appearance of patient’s smile, making it essential to carefully evaluate the underlying causes before deciding on treatment [5].

Tooth discolorations are generally classified as either extrinsic or intrinsic [6]. Extrinsic discolorations occur due to the accumulation of chromogenic substances on the external surface of the tooth, and can often be removed by professional tooth brushing.

Intrinsic discolorations occur as a result of changes in the structural composition or thickness of the tooth substrates. Treatment of intrinsic discoloration typically involves various tooth whitening techniques [7]. The effectiveness of peroxide for whitening vital teeth was discovered in the 1960s when Klusmier was using a gel containing carbamide peroxide to treat periodontic inflammation after treatment [6]. However, it was not widely taken up until the 1980s.

In the 21st century, demand for tooth whitening increased even further, and clinicians and scientists developed minimally invasive approaches that would function as effective alternatives to placing veneers or crowns on otherwise healthy teeth [8]. Today, dental whitening techniques are generally classified into vital and non-vital tooth whitening. This study is concerned with vital tooth whitening techniques, which are further subdivided into three categories: (1) in-office whitening; (2) at-home bleaching without professional supervision, this typically involving over-the-counter products; and (3) at-home whitening supervised by a dentist [9].

In-office dental whiting typically involves the use of high-concentration hydrogen peroxide whitening agents, containing 25–40% hydrogen peroxide [10]. While these procedures have a strong bleaching effect, they must be performed in the clinic, and there is a risk of oversensitivity and pain during the procedure.

At-home bleaching without professional supervision, known as over-the-counter (OTC) whitening, involves the use of products containing low concentrations of whitening agents, which are purchased and applied without professional supervision [11]. As these products must be formulated to be safe when used without any supervision, they have low effectiveness and are designed to be used over a long period, for a gradual improvement in tooth color.

At-home dental whitening with supervision by the dentist is a compromise between these two options. The direct guidance to the patient makes it safe to use higher concentrations of whitening agents, and custom trays can be prepared to optimize the exposure of the tooth surface to the active ingredients.

The standard version of this technique involved the overnight use of 10% carbamide peroxide in custom-fitted trays. Studies have shown that the use of this technique nightly, for eight to ten hours per night, over a period of two or three weeks produces whitening equivalent, or superior, to that achieved by in-office whitening performed at weekly appointments over the same period [12].

This treatment is effective, reduces chair time, and the lower concentration of peroxide means that oversensitivity is a less common concern. However, the success of this method relies heavily on patient compliance, as the outcome is closely tied to how diligently the instructions are followed. Tooth sensitivity and soft tissue irritation, particularly with excessive or prolonged use of the whitening agents, can still be a concern. There is still demand for faster and more effective products.

This demand has led to the development of at-home products that act more quickly and can be used during the day for a shorter period of time. Initially, this required two to four hours, but as longer whitening periods, particularly during the day, reduce patient compliance, efforts have been made to develop products that can be used over shorter periods of 60 to 90 min. This includes both hydrogen peroxide-based and carbamide peroxide-based products. The hydrogen peroxide based products use a concentration of 6%, while those based on carbamide peroxide use a concentration of 16%.

As these concentrations fall between those of earlier products, there has been little work directly assessing the effectiveness of these products. This paper provides a preliminary assessment and comparison of the efficacy of two products using 6% hydrogen peroxide, and one using 16% carbamide peroxide.

## 2. Materials and Methods

The fundamental whitening capacity of three dentist-supervised at-home whitening agents was compared using the eggshell bleaching technique.

The three materials compared were TiON Take Home with (GC, Tokyo, Japan), Opalescence Go (Ultradent, South Jordan, UT, USA), and Angelus Home 16% (Angelus, Londrina, Paraná, Brazil). TiON Take Home with and Opalescence Go include 6% hydrogen peroxide, while Angelus Home 16% includes 16% carbamide peroxide. Full details of the materials are presented in Table 1.

**Figure 1:**
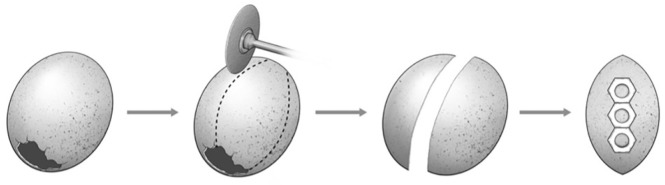
Experimental set-up. A section was cut from a cleaned eggshell, and patches of the tray material were affixed. Holes in the tray material were created wells for the whitening agent, and the area within which color changes were measured was defined.

Brown eggshells were prepared by emptying and cleaning brown hens’ eggs (Yōdo Eggs HikariNosan Corporation, Yokohama, Japan) and cutting 120° sections from the shells, as shown in Figure 1. The material used to make the trays for each whitening agent was cut into a strip, and two-sided adhesive tape applied to one side. Circular holes 5 mm in diameter were created in the material using a hole-punch, and the strip cut into individual pieces, each containing a single hole. The pieces were then affixed to the eggshell. Three pieces were used for each treatment regime (*n* = 3).

**Figure 2:**
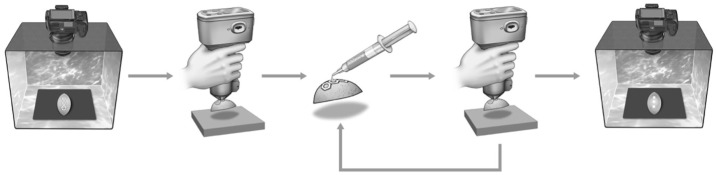
Color measurement method. The eggshell was photographed for reference at the beginning and end of the experiment. The color of each treatment area was measured with a spectrophotometer before treatment and after 3, 5, 7, and 10 applications. For Angelus Home 16%, the color was also measured after 12 and 14 applications. The eggshell was photographed again for reference at the end of the experiment.

The samples were then placed on a standard black background in a light box and photographed with a single-lens reflex digital camera (Canon, Tokyo, Japan). They were removed from the light box, and the color of the shell was measured against and standard black background using a spectrophotometer (CM-700d, Konika Minolta, Tokyo, Japan) (Figure 2).

The tray material is about 1 mm thick, which creates a depression that can contain the whitening agent. The whitening agent was placed into the hole in the tray material and covered with transparent OHP film (A-One OHP Film, 3M Japan, Tokyo, Japan). The sample was kept at 37 °C for the treatment period to simulate intra-oral conditions. Each whitening agent was applied for the length of time recommended by the manufacturer: 60 min (W60) or 90 min (W90) for TiON Take Home with and 90 min with both Opalescence Go (G90) and Angelus Home 16% (A90).

This was repeated a number of times based on the recommended number of days of treatment for each agent, assuming one application per day: 10 times for TiON Take Home with and Opalescence Go, and 14 times for Angelus Home 16%.

The color of the shell was measured with the spectrophotometer after the 3rd, 5th, 7th, and 10th treatments and also after the 12th and 14th treatments with Angelus Home 16%. After all treatments had been completed, the eggshells were photographed again under the same conditions as at the beginning of the experiment.

After confirming normality, postoperative color differences were analyzed using one-way analysis of variance (ANOVA), followed by Tukey’s multiple comparison test (*p* < 0.05).

## 3. Results

### 3.1. In Vitro Results

**Figure 3:**
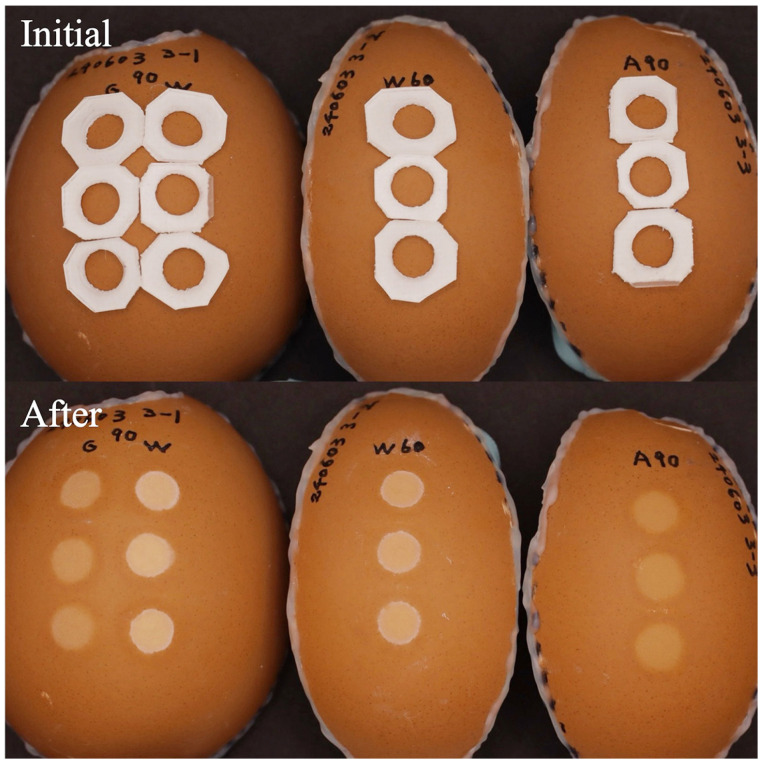
Photographs of the specimens before and after treatment with the whitening agent.

The photographs of the samples before and after treatment are shown in Figure 3.

**Figure 4:**
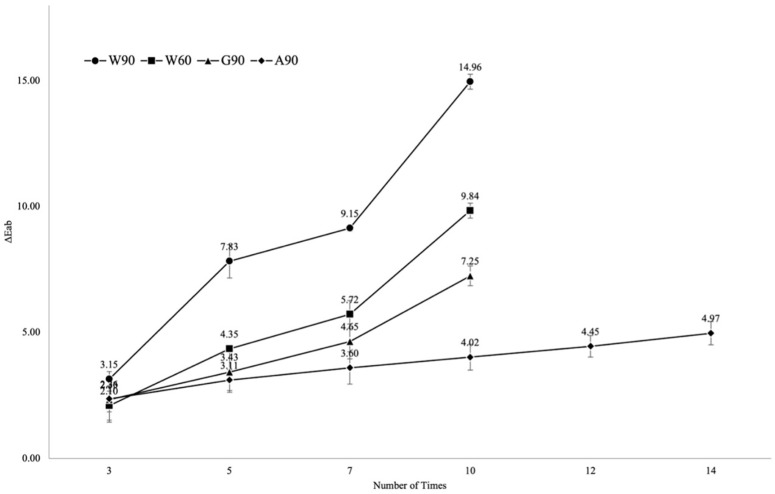
Whitening results. A ∆E*_ab_ of 1.5–3.0 indicates a noticeable change in color, 3.0–6.0 an appreciable change, 6.0–12.0 indicates considerable change, and over 12.0 indicates more considerable change. A90: Angelus Home 16%, 90 min application period; G90: Opalescence Go, 90 min application period; W60: TiON Take Home with, 60 min application period; W90: TiON Take Home with, 90 min application period. Error bars indicate standard deviation.

The results for ∆E*_ab_ are shown in Figure 4 and Table 2. A ∆E*_ab_ of 1.5–3.0 indicates a noticeable change in color, 3.0–6.0 an appreciable change, 6.0–12.0 indicates considerable change, and over 12.0 indicates more considerable change.

All the whitening agents show a similar trend in ∆E*_ab_, with a monotonic increase in color change that is relatively large over the first three applications, and then continues at a roughly constant rate over the subsequent applications. The rates are quite different, however, with Angelus Home 16% showing an average change in ∆E*_ab_ of 0.24 per application, Opalescence Go showing an average change of 0.70, and TiON Take Home with showing an average change of 1.1 with 60 min periods and 1.7 with 90 min periods.

The individual changes in L*, a*, and b* also show similar trends for most of the materials, with an increase in L* and a decrease in a* and b*. However, A90 shows an increase in b*, unlike the other materials.

By the end of the application period, all the whitening agents had achieved at least an appreciable change, while Opalescence Go and TiON Take Home with at 60 min application periods achieved much change. TiON Take Home with at 90 min application periods achieved considerable change after 5 applications and more considerable change after 10.

Significant differences were observed among all groups (W90, W60, G90, and A90), with all pairwise comparisons showing statistical significance (Tukey’s test, *p* < 0.05).

### 3.2. Case Study

The patient was a 21-year-old female who presented at our clinic with an interest in whitening her teeth. No caries was detected in the anterior teeth, and the periodontal condition was good. The patient was a student, and her studies made it difficult for her to visit the clinic frequently, and so, it was decided to use TiON Take Home with as the whitening agent. This uses custom trays, with 6% hydrogen peroxide as the active ingredient, and is marketed as being effective with short application periods and a shorter treatment cycle.

Full scaling and PMTC were carried out, and the pretreatment shade of the teeth was measured with a dental spectrophotometer (Optishade Styleitaliano, Smileline, Saint-Imier, Switzerland). On the upper right central incisor, the following values were measured: L: 77.8; a: 4.2; b: 15.1. To prepare the custom tray, a detailed impression was taken with a super-hydrophilic silicone putty (Fusion II, GC), a working model was created, and the tray sheet (TiON Home Tray Sheet, GC) was pressure fitted to the model. The tray design was scalloped at the incisal end and extended 1 mm gingivally at the gingival margin. Whitening agent was painted on to the labial and buccal sides of the tray, and it was fitted to the teeth. The patient was instructed to apply the tray for 60 min once per day over a period of ten days. Furthermore, in order to treat white spots, the patient was directed to not only conduct whitening, but also to apply mineralizing paste (post-whitening treatment paste, GC) after treatment, to supplement the mineral content.

**Figure 5:**
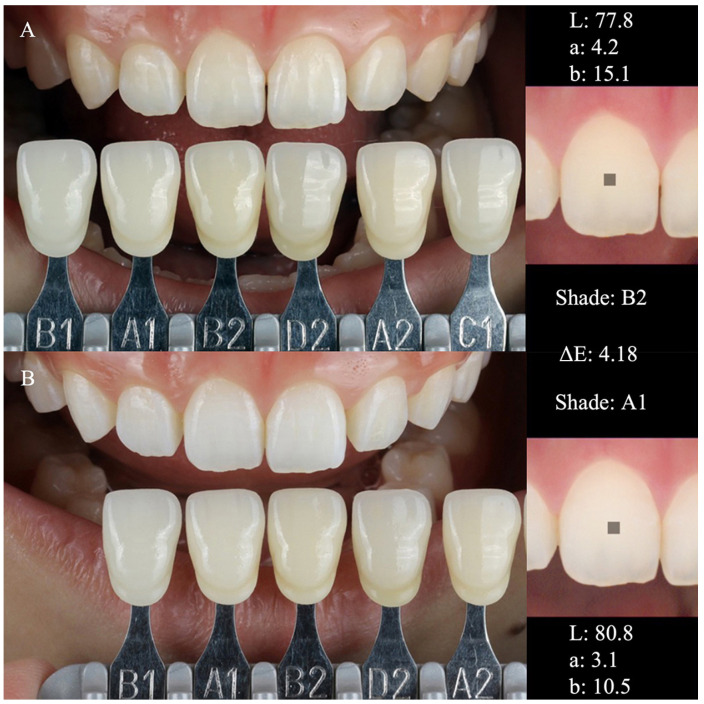
Facial view of upper and lower teeth before and after whitening treatment. (**A**): Vitashade guide showing shade B2 for right central incisor as determined by spectrophotometer measurement before whitening treatment; (**B**): Vitashade guide showing shade A1 for right central incisor as determined by spectrophotometer measurement after whitening treatment. L, a and b indicate the components of the spectrophotometric measurement of the color. ∆E indicates the change in color.

The results of color measurements of the upper right central incisor after 10 treatment cycles were as follows: L: 80.8; a: 3.1; b: 10.5. The ∆E was 4.18, which greatly exceeds the JIS standard for whitening of 2.0, and is easily perceptible even by untrained individuals (Figure 5).

While it is not possible to generalize from the result of this single case, the clinical results are consistent with the in vitro results.

## 4. Discussion

The use of brown chicken eggshell as a standard material for in vitro measurements of bleaching efficacy was proposed by Ikemi et al. [13] and is now a standard approach. While the absolute values of color change are not applicable to the clinical situation, it remains a valuable approach for the comparison of multiple materials. In particular, it allows a high level of standardization across the samples, which permits a comparison of the materials under extremely similar conditions, without the influence of differences in the trays or application methods used in the clinic.

In this study, the color change was analyzed quantitatively using spectrophotometry. The photographs taken before and after treatment serve as a visual, qualitative check on the quantitative results. The differences between the different treatments are clearly visible in Figure 3, and this provides further support for the quantitative results.

It has recently become more common to use ∆E_00_ in reporting color changes in dental contexts, as this measure has been developed to more closely reflect human color perception. However, this study aimed to compare the intrinsic color changes in brown eggshells, and for this purpose, ∆E*ab is more suitable, as it is a more direct measurement of color changes. ∆E*ab was used for the clinical case as well to ensure comparability between the results. In this case, there were clear changes in color, and so, the choice of measurement is not expected to make an important difference to the results.

The increase in b* after bleaching with A90 is anomalous, and the results of this experiment do not permit a grounded hypothesis as to the reason. It may be significant that A90 is the only bleaching agent using carbamide peroxide, but the absence of comparison agents using the same active ingredient makes it impossible to say whether this is the reason.

To date, the standard active ingredient in home whitening kits has been carbamide peroxide, and concentrations of up to 40% have been available on the market. On the other hand, the gold standard for home whitening kits was 10% carbamide peroxide. This was because, although higher concentrations have higher whitening effectiveness, they also increase the risk of oversensitivity and may be regulated differently in different countries. However, if 10% carbamide peroxide is used, the protocol requires at least 120 minutes’ use of the tray, and a single cycle is two weeks. Current demands call for protocols with shorter application periods and shorter cycles. Thus, the frequency of use of 6% hydrogen peroxide as an active ingredient has become higher, as it can be expected to achieve whitening more quickly in both respects. In both cases, the ultimate active ingredient is hydrogen peroxide, which is produced by breakdown of carbamide peroxide. A simple comparison of the molar content of hydrogen peroxide suggests that 6% hydrogen peroxide will have 1.8× the whitening effect of 10% carbamide peroxide, while the JIS standard asserts that a concentration of hydrogen peroxide equal to 0.36 of the concentration of carbamide peroxide has the same whitening effect [14].

For this reason, we chose Angelus Home 16% as the carbamide-based comparison material, because based on the JIS standard [14], this would be expected to have approximately the same whitening effect as 6% hydrogen peroxide. However, the whitening effect is not a simple result of the concentration of hydrogen peroxide and is also strongly influenced by the composition of the whitening agent, as we can see from the results of this experiment. A fundamental difference between hydrogen peroxide and carbamide peroxide is that carbamide peroxide must break down to carbamide and hydrogen peroxide before it can begin bleaching, and therefore, carbamide peroxide is at a disadvantage when short application periods are required. As effective levels of hydrogen peroxide were not measured in this experiment, it is not possible to confirm that the bleaching periods used with these products are short enough to be an issue, but it is a possibility. In such a case, the effective concentration of hydrogen peroxide may be even lower than the calculation suggests, as some is still bound up in carbamide peroxide when the whitening agent is removed from the teeth. This is one possible explanation for why, in this experiment, the whitening achieved by hydrogen-peroxide-based agents in one week was only achieved after two weeks by the carbamide-peroxide-based agent.

However, speed of whitening is not the only factor of importance. Dehydration of the tooth surface is thought to be an important factor in the development of oversensitivity, and it can be speculated that the presence of carbamide peroxide might inhibit dehydration, or at least promote it less than simple hydrogen peroxide. On the other hand, reducing the application period and the length of the treatment cycle are also thought to be ways to reduce the risk of oversensitivity. While these factors were not measured in this experiment, they would need to be taken into account in a full clinical recommendation. It is therefore necessary to consider all aspects of the treatment when deciding on the best approach to a particular case.

For example, if fast whitening results are the highest priority, on the basis of these results, TiON Take Home with might be the best choice. On the other hand, in a case in which there is concern about oversensitivity arising from dehydration of the teeth, Angelus Home 16%, which uses carbamide peroxide, might be the recommendation. Finally, if it is important to avoid taking an impression, then Opalescence Go, which uses standard trays, would be a strong candidate.

These results also show a clear difference between the two systems using hydrogen peroxide, Opalescence Go and TiON Take Home with. Opalescence Go at an application period of 90 min caused a color change comparable to that resulting from TiON Take Home with at an application period of 60 min, and clearly smaller than that observed with TiON at an application period of 90 min. As the two systems have equal concentrations of hydrogen peroxide as the whitening agent, other differences in the composition must be responsible for the disparity in effect. A range of factors can affect this, such as the hydrophilicity of the gel supporting the whitening agent, and the ease with which the agent is released by the gel [15]. Such differences are to be expected in this case, as Opalescence Go is formulated for use with disposable pre-filled trays, while TiON Take Home with is formulated for use with custom trays. However, these factors were not measured in this study, and the manufacturers provide little information about the composition, as such information is proprietary. It is not therefore not possible to determine the causes of the differences in performance.

There are several limitations to these results. The most important is that it only considers three test samples for each material and a single clinical case. This is a preliminary exploration of the comparative efficacy of these materials, illustrated by a clinical case using one of them. While it suggests that these materials may be effective, and that further studies with larger sample sizes and a wider range of data would be valuable, it should not be used as a basis for clinical recommendations.

As a further consequence of the small number of samples, the statistical measures should be treated cautiously. They are as illustrative and exploratory as the rest of the study, and studies with larger sample sizes would be valuable to confirm their robustness.

Another limitation is that the use of brown chicken eggshells as a standard for measuring whitening efficacy still needs wider confirmation of its clinical implications. While it is an effective way to compare materials, there is still limited information on what the results mean directly for clinical efficacy. Furthermore, the single clinical case study discussed in this paper can only be considered as illustrative. While it is consistent with the results of the in vitro testing, it cannot be said to confirm their clinical applicability. Further studies using teeth or dental enamel would be particularly valuable.

Finally, as hydrogen peroxide is an irritant and can cause damage to tissues, the regulations for its use differ between jurisdictions. In the USA, home whitening systems employing overnight application of hydrogen peroxide are available, while they are not permitted in Japan. This means that research adapted to the materials and techniques available in each region is of great importance, and that systematic reviews must take this into account when making recommendations for practice. This study looks only at systems available for use in Japan, and while such studies are essential, it is also important to study the systems available in other countries.

## 5. Conclusions

This exploratory study found clear differences in whitening efficacy between different home whitening systems, which are not entirely attributable to differences in the active whitening ingredient. All three systems were effective, and further research on their appropriate clinical use would be valuable.

## Figures and Tables

**Table 1:** Whitening materials used in this study.

Materials	Ingredients	Manufacturer	Lot
TiON Take Home with	6% Hydrogen peroxide Polyhydric alcohol Viscosity modifier pH adjuster	GC	2403061
Opalescence Go	6% Hydrogen peroxide Purified water Glycerin Carboxyvinyl polymer Silicon dioxide pH adjuster	Ultradent	BWDN2
Angelus Home 16%	16% carbamide peroxide Glycerin Polyacrylic acid Fragrance, etc. Potassium nitrate Sodium fluoride	Angelus	66378

**Table 2:** Result of color difference (ΔEab).

		Initial	After	ΔEab
W90	L*	46.50	60.38	14.96 (0.30) ^a^
	a*	11.14	7.45	
	b*	22.29	18.15	
W60	L*	47.26	56.73	9.84 (0.30) ^b^
	a*	11.06	9.46	
	b*	22.69	20.63	
G90	L*	46.45	53.35	7.25 (0.39) ^c^
	a*	11.40	9.64	
	b*	23.00	21.81	
A90	L*	47.89	52.08	4.97 (0.46) ^d^
	a*	10.96	10.14	
	b*	23.17	25.69	

Values in parentheses are standard deviations. Different lowercase letters indicate statistically significant differences (*p* < 0.05).

## Data Availability

The original contributions presented in this study are included in the article. Further inquiries can be directed to the corresponding author.
